# Supplementary material for: Identification of photoperiod-induced specific miRNAs in the adrenal glands of Sunite sheep (Ovis aries)
Source: Front Vet Sci. 2022 Jul 22;9:888207. doi: 10.3389/fvets.2022.888207 (PMC9354845; doi:10.3389/fvets.2022.888207)
Supplement: Supplementary file 1 [file Data_Sheet_1.ZIP › Supplementary file/miRDeep2 file.zip!/miRDeep2 file/Know_Structure/SP42A1/oar-let-7d.pdf]

miRBase precursor : oar-let-7d
Total read count : 17673
oar-let-7d read count : 16208
remaining reads : 1465

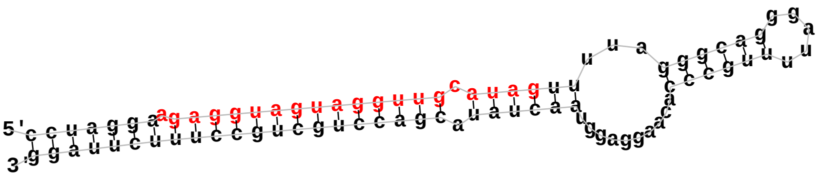

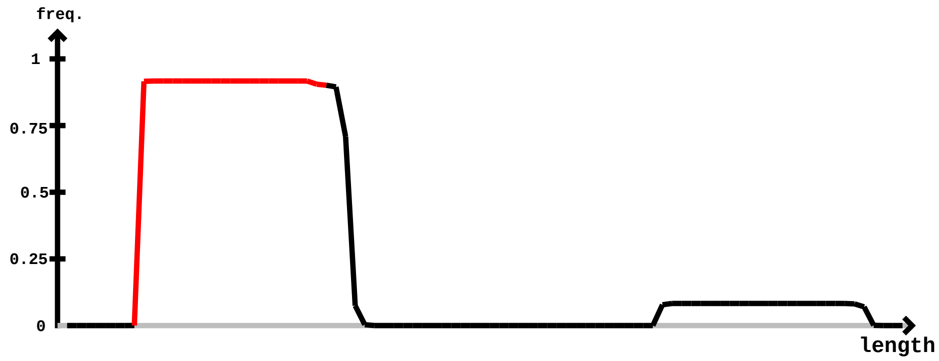


```
        oar-let-7d
5'- ccuaggaagagguaguagguugcauaguuuuagggcagggauuuugcccacaaggagguaacuauacgaccugcugccuuucuuagg   -3'    exp
    (((((((.((((((((((((((.((((((...((((((.....)))))).........)))))).))))))))))))))))))))))   reads      mm       sample
    ......aagagguaguagguugcauagu...........................................................   3          0        seq
    ......aagagguaguagguugcauaguu..........................................................   1          0        seq
    .......agagguaguagguugca...............................................................   5          0        seq
    .......agagguaguagguugcau..............................................................   210        0        seq
    .......agagguaguagguugcaua.............................................................   67         0        seq
    .......agagguaguagguugcauag............................................................   111        0        seq
    .......agagguaguagguugcauagu...........................................................   3277       0        seq
    .......agagguaguagguugcauaguu..........................................................   11200      0        seq
    .......agagguaguagguugcauaguuu.........................................................   1255       0        seq
    .......agagguaguagguugcauaguuuu........................................................   58         0        seq
    ........gagguaguagguugcauagu...........................................................   4          0        seq
    ........gagguaguagguugcauaguu..........................................................   13         0        seq
    ........gagguaguagguugcauaguuu.........................................................   1          0        seq
    .........agguaguagguugcauaguu..........................................................   3          0        seq
    .............................................................cuauacgaccugcugccuu.......   1          0        seq
    .............................................................cuauacgaccugcugccuuu......   26         0        seq
    .............................................................cuauacgaccugcugccuuuc.....   181        0        seq
    .............................................................cuauacgaccugcugccuuucu....   1160       0        seq
    .............................................................cuauacgaccugcugccuuucuu...   10         0        seq
    ..............................................................uauacgaccugcugccuuu......   2          0        seq
    ..............................................................uauacgaccugcugccuuuc.....   14         0        seq
    ..............................................................uauacgaccugcugccuuucu....   67         0        seq
    ..............................................................uauacgaccugcugccuuucuu...   4          0        seq
```
